# Comprehensive Survey on Improved Focality and Penetration Depth of Transcranial Magnetic Stimulation Employing Multi-Coil Arrays

**DOI:** 10.3390/ijerph14111388

**Published:** 2017-11-14

**Authors:** Xile Wei, Yao Li, Meili Lu, Jiang Wang, Guosheng Yi

**Affiliations:** 1 Tianjin Key Laboratory of Process Measurement and Control, School of Electrical and Information Engineering, Tianjin University, Tianjin 300072, China; xilewei@tju.edu.cn (X.W.); lucklyt@163.com (Y.L.); jiangwang@tju.edu.cn (J.W.); 2 School of Information Technology Engineering, Tianjin University of Technology and Education, Tianjin 300222, China; meililu@tute.edu.cn

**Keywords:** target location, transcranial magnetic stimulation, electric field distribution, multi-coil array

## Abstract

Multi-coil arrays applied in transcranial magnetic stimulation (TMS) are proposed to accurately stimulate brain tissues and modulate neural activities by an induced electric field (EF). Composed of numerous independently driven coils, a multi-coil array has alternative energizing strategies to evoke EFs targeting at different cerebral regions. To improve the locating resolution and the stimulating focality, we need to fully understand the variation properties of induced EFs and the quantitative control method of the spatial arrangement of activating coils, both of which unfortunately are still unclear. In this paper, a comprehensive analysis of EF properties was performed based on multi-coil arrays. Four types of planar multi-coil arrays were used to study the relationship between the spatial distribution of EFs and the structure of stimuli coils. By changing coil-driven strategies in a basic 16-coil array, we find that an EF induced by compactly distributed coils decays faster than that induced by dispersedly distributed coils, but the former has an advantage over the latter in terms of the activated brain volume. Simulation results also indicate that the attenuation rate of an EF induced by the 36-coil dense array is 3 times and 1.5 times greater than those induced by the 9-coil array and the 16-coil array, respectively. The EF evoked by the 36-coil dispense array has the slowest decay rate. This result demonstrates that larger multi-coil arrays, compared to smaller ones, activate deeper brain tissues at the expense of decreased focality. A further study on activating a specific field of a prescribed shape and size was conducted based on EF variation. Accurate target location was achieved with a 64-coil array 18 mm in diameter. A comparison between the figure-8 coil, the planar array, and the cap-formed array was made and demonstrates an improvement of multi-coil configurations in the penetration depth and the focality. These findings suggest that there is a tradeoff between attenuation rate and focality in the application of multi-coil arrays. Coil-energizing strategies and array dimensions should be based on an adequate evaluation of these two important demands and the topological structure of target tissues.

## 1. Introduction

Transcranial magnetic stimulation (TMS) is currently regarded as a noninvasive treatment that holds great promise to be used in basic and clinical neurophysiology [1,2,3,4]. TMS uses brief, repetitive pulses in a stimulating coil configuration located near the scalp to evoke electric fields (EFs) and eddy currents in the conductive brain tissue [5]. By altering the neural transmembrane potential, TMS can modify the cortical excitability and modulate neural activities [5,6,7,8,9]. Conventional TMS devices mainly stimulate brain regions limited to cortical sites, whereas the deeper cerebral tissues can be stimulated only by a transsynaptic effect exerted by the activated superficial parts [1].

Modulation of the deep brain structures demand a large EF to be acutely focused on interested tissues deep in the cerebrum. Unfortunately, EFs evoked by common circular coils diffuse and decay rapidly as distance from the cortex increases [10]. The figure-8 coil, designed for a better focusing ability [11], usually activates an EF normal to the cortical tissue, which may lead to painful muscle contractions [12,13,14,15]. Hence, the “H-coil” and an improved coil system consisting of a Halo coil together with a circular coil were proposed, which increased the penetration depth of the target region but failed to make significant improvements in field focus [16,17,18]. To bypass the limitations of these devices and locate the target sites in TMS treatment efficiently, multi-coil arrays have been designed for a more concentrated and deeply penetrated field [10,19,20]. Compared to other stimulating coil configurations, multi-coil arrays improve the cortical activating efficiency [21]. Furthermore, this kind of equipment is composed of numerous independently driven coils, which makes modulating the EF distribution possible by changing the energizing strategy. Previous studies have evaluated multichannel TMS systems by analyzing field concentration based on realistic stimulations [22] and have been devoted to finding optimal methods for designing and implementing TMS coil arrays [10,23,24]. However, the target tissues selected to complete the optimization are limited in the cortical surface, and there is insufficient demonstration of how an EF induced by a multi-coil array is accurately controlled by the spatial arrangement of stimulating coils. Although a stimulated volume and field penetration depth have been comprehensively considered in some studies [10,15], the maximum depth of the activated region has never been more than 4 cm, which means that information regarding EF distribution properties over the brain model is insufficient. Theoretical studies have focused on the selection of driving currents and coil orientations for stimulating required fields [10,25,26], but the reports based on complex mathematical algorithms have not appeared to have shown energizing strategies to fulfill the demands for various target shapes and sizes. In order to take full advantage of multi-coil arrays by clarifying the relevance between EF distribution and the spatial geometry of stimuli coils, strength attenuation and the focality of EF induced by multi-coil arrays in large-scale cerebral structures were quantitatively studied. Using a spherical head model, we constructed seven coil combinations of diverse energizing strategies based on a 16-coil square array. Furthermore, we used four types of planar array of distinct dimensions and coil numbers to demonstrate the tradeoff between attenuation rate and focality of this configuration. Since there is insufficient knowledge of locating the target sites, which has long been an important issue in TMS involving therapeutic safety and efficiency [19], we segmented the upper half of the brain cortex into 50 units to evaluate the control of stimulation. By comparing the expected interested units with the actual activated regions evoked by specific coil-driven strategies, we found that a 64-coil array with an edge length of 159 mm and a coil outer radius of 9 mm can precisely locate the cortical region within an area of a single unit. Comparison of a figure-8 coil, a planar array, and a cap-formed array demonstrates that these three types of coils have similar attenuation rates of EF intensity, but multi-coil arrays perform better in target location, indicating an improvement in stimulating focality.

## 2. Materials and Methods 

### 2.1. Computational Models

**Figure 1:**
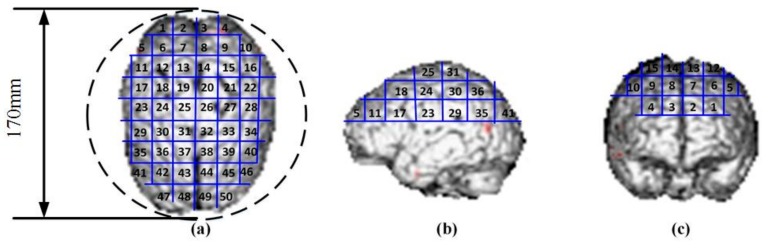
Segmentation method on the surface layer of top half of the head model. (**a**–**c**) Different orientations of observation.

We used a homogeneous spherical head model with an isotropic conductivity of 0.33 S/m 170 mm in diameter. This data is consistent with the realistic conductivity of scalp and cortex, which occupy a large proportion of the brain. Various tissue layers (scalp, skull, corticospinal fluid, and brain) were not differentiated since magnetically induced EF in a sphere is insensitive to radial variations of conductivity [27,28,29]. Tissue conductivities have small quantitative effects on the variation properties of the induced EF [30], but it is inadequate to influence the selection of coil spatial arrangement. Furthermore, results extracted from a spherical model are not limited to the anatomy of a specific subject, allowing for general conclusions on the application of coil configurations. Although there are some differences between spherical and realistic models, many important features are common to these two methods, supporting the study of EF properties determined by the spatial arrangement of coils [31]. Moreover, the spherical head model provides a standardized platform to evaluate and compare coil designs [29]. To quantitatively describe the regions activated by multi-coil arrays and to test the accuracy of the target location, the cortex of the spherical head model was segmented into 50 units that correspond to realistic head dimensions, which is shown in Figure 1. As the spherical head model is 170 mm in diameter, each single unit is an approximately 19 mm^2^ square. Target regions of various shapes and sizes can be clearly depicted by the segmented head model.

**Figure 2:**
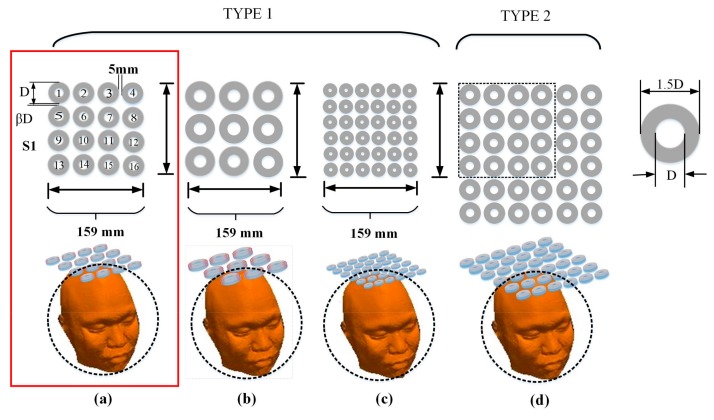
(**a**) The 16-coil array is shown in the front view, where Figures 1–16 are used to distinguish different coils. Directions of stimulating current are marked out by arc arrows. To simplify the calculation process, we used D to denote the outer diameter of individual coil and βD to represent the minimum distance between adjacent coils in a row. The models in (**b**–**d**) are those of the 9-coil array, the 36-coil (dense) array, and the 36-coil (disperse) array, respectively. Ratio between of the outer diameter of the coil to the minimum coil–coil distance was fixed at 0.55, but the total numbers of coils in the arrays are distinct.

**Figure 3:**
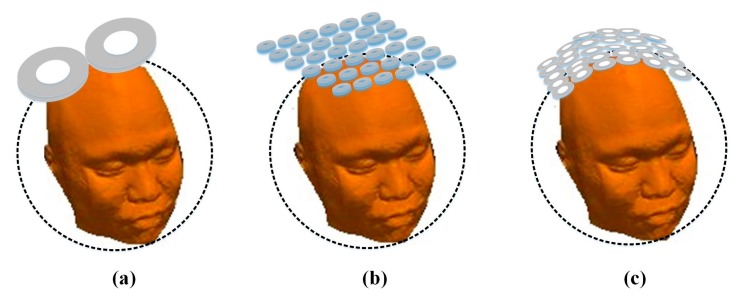
Coil arrays used for comparison. (**a**) figure-8 coil; (**b**) 36-coil planar array; (**c**) 36-coil cap-formed array.

Four types of planar multi-coil arrays, a typical figure-8 coil, and a 36-coil cap-formed array were used to analyze spatial EF properties and make further comparisons. The four types of planar multi-coil arrays with diverse coil number and dimension are shown in Figure 2. Coil arrays in Figure 2a–c have an identical side length of 159 mm. Each individual coil consists of two layers of windings and the wire in simulation is 0.75 mm in diameter. The outer radii of cylindrical coils in Figure 2a–c are 18 mm, 24.5 mm, and 11.9 mm with corresponding winding turns of 16, 22, and 12, respectively. The array in Figure 2d has the same coil number as that in Figure 2c, but has a larger individual stimulating coil. The ratio of the coil–coil interval distance to the outer radius of an individual coil is fixed at 0.55. The minimum spacing between the coil planar and the surface of head model is 5 mm to account for the thickness of the coil insulation, which is not included in the coil models [32,33]. The typical figure-8 coil, the 36-coil planar array, and the 36-coil cap-formed array are depicted in Figure 3. The figure-8 coil model was on the basis of a standard Magstim 70 mm diameter coil, as shown in [34].

### 2.2. Coil Combinations of Diverse Energizing Strategies

**Figure 4:**
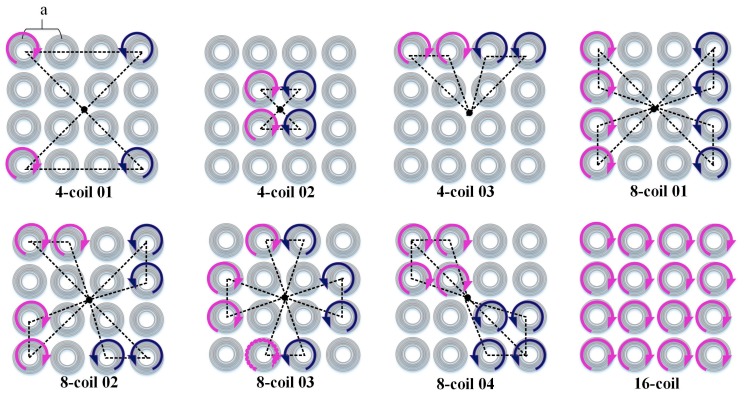
Sketch maps of different coil combinations based on the 16-coil array. Current directions are indicated by arrows. Coils with clockwise or anticlockwise current are regarded as stimulating coils, whereas the gray ones represent coils without electricity. “a” indicates the length between two adjacent coils and the connecting lines in each coil array mark out the specific areas corresponding to different energized coil combinations.

Since the structure of stimulating coils plays a crucial part in determining the depth and focality of activation, we first focused on the energizing strategy of coils in a planar array to take full advantage of the control flexibility and develop a procedure of optimizing sets of stimuli coils. Here, the spatial arrangement of stimulating coils reflects the shape and size of a specific combination. Reports have demonstrated that a figure-8 coil composed of two sets of adjoining circular loops can induce a concentrated EF beneath the coil center [11]. Based on this, we aimed to study where the specific region activated by multiple stimulating coils is located in the brain and how the EFs evoked by coils of complex spatial arrangement superposes with each other. We assumed that the relative position and distance of energized coils are important factors, which significantly affect the induced EF distribution. To model various combinations and maintain the diversity of spatial arrangements, we chose the 4-coil group and the 8-coil group as objects of our study. The sketch maps of coil combinations are shown in Figure 4. The effect of coil–coil distance can be demonstrated by 4-coil 01 and 4-coil 02, both of which have an identical coil number but distinct spatial structures. 4-coil 03 can be regarded as an atypical figure-8 coil, whose loop of each side is replaced by two energized coils with the same stimulating current. Besides, 8-coil 01, 8-coil 02, and 8-coil 03 have analogous symmetry and energizing strategies in general, while distinctions of these combinations are reflected in relative positions of individual stimulating coils. Coils in 8-coil 04 are compactly distributed, but still have a desirable comparability to 4-coil 02 and 4-coil 03. In order to quantify the arrangement of coils with a unified standard, we proposed a concept of a compact degree (CD), which can be depicted by a simple formula as follows.

(1) $$CD=\frac{1}{S•T}$$

where S represents the smallest area encircled by connecting lines that link the planar array center with each single center of the energized coils, as is shown in Figure 4. T indicates the total length of center–center joining lines. The relative position and distance of stimulating coils can be depicted by S and T, which depict the distinctions of coil combinations quantitatively. To simplify the calculation process, the core–core length of adjacent coils in the same row is represented by “a.” A coil combination of a larger value of CD has a more compactly distributed structure. Seven coil combinations and the corresponding CD values are listed in Table 1. These combinations are classified into two types: the 4-coil group and the 8-coil group, where the figures indicate the energized coils. We can compare the ability of each coil to reach deep brain structures and evaluate the attenuation profile of EF peak intensity along the direction passing through brain center at different distances ranging from 1 to 7 cm.

### 2.3. Numerical Simulations

EFs induced by different types of TMS coil configurations were determined using the finite element method (FEM). Simulations in this work were implemented in ANSYS 15.0 (ANSYS, Pittsburgh, PA, USA). Transient calculation was used and selected coils were energized by a monophasic current pulse of a duration of 250 μs. We applied the different current density as a controllable input variable, which is constrained under $2.27\times 10^{6}A/m^{2}$ (1A in a single wire). Biophysics of TMS depends on the electrical features of the nervous tissues. In particular, TMS performance obeys Faraday’s law of induction, which states that a closed electrical circuit Σ within an enclosed magnetic field B, whose flux is time-dependent, will be subject to a variation in the electrical current I induced by the magnetic field itself [35]. This law can be expressed as follows:

(2) $$\oint_{\partial \Sigma }Ids=-\frac{d\Phi_{B}}{dt}$$

where $\Phi_{B}$ is the flux of the magnetic field. Equation (2) also shows that the resulting electrical current propagates in the opposite direction to the variation in the magnetic field flux. The presence of a dynamic magnetic field leads to a different variation tendency in the intensity of the electric field of a closed loop electrical system. The magnetic field strength B(r,t) obeys the Biot-Savart law, which is shown in Equation (3):

(3) $$B(r,t)=\frac{\mu_{0}}{4\pi }I(t)\oint_{c}\frac{dI(r\prime )\times (r\prime -r)}{|r-r\prime {|}^{3}}.$$

The induced EF considerably attenuates in strength as it penetrates into deep tissues of the brain. This is mainly due to the characteristics of the cerebral tissue, acting as a conductor when both the air and the skull can be regarded as insulators. As a result, besides the field $E_{I}$ induced by the coil configuration, a concentration of electric charges on the cortical surface can also generate a second field $E_{B}$ with a direction opposite to $E_{I}$. The total EF induced in the head by stimulating coils is the superposition of the two field vectors [36]:

(4) $$E_{TOT}=E_{I}+E_{B}.$$

## 3. Results

### 3.1. Electric Field Attenuation Rate

**Figure 5:**
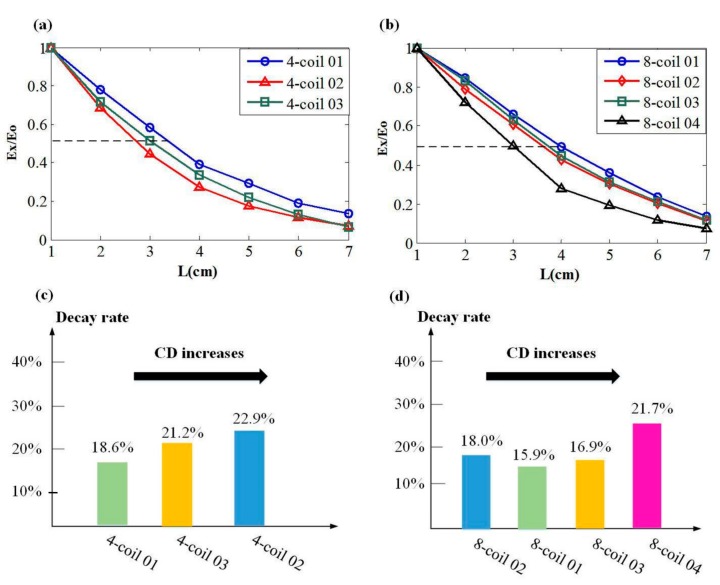
Relationship between the electric field (EF) intensity and the depth of specific brain regions. (**a**) The intensity attenuation profiles of 4-coil combinations. Here, **L** indicates the distance from the surface of the head model along the direction passing through the brain center. E_X_ represents the EF intensity of a targeted depth, and E_O_ is the value of cortical EF intensity. (**b**) The intensity attenuation profiles of 8-coil combinations. (**c**) Relationship between the decay rate of induced EF strength and the value of CD among the 4-coil group. (**d**) Relationship between decay rate of field intensity and the CD level of 8-coil group.

Previous studies have shown that the induced EF intensity is largely related to the distance between coil configurations [21,22,37] and the targeted tissue. A slow attenuation rate of induced EF generally typifies a large penetration depth and activated range. However, there is no report that focuses on the spatial arrangement of devices with the same coil number but different coil distribution modes. Thus, the superposition of the induced EF and the spatial distribution of stimulating coils have not been fully characterized. To assess and present the variation rules of the EF, we focused on evaluating and analyzing the attenuation rate of EF intensity. **L** indicates the distance from the surface of the head model along the direction passing through the brain center. Peak values of EF intensity at different **L** values are normalized with respect to the maximum of the EF amplitude distribution estimated on the cortex and are quantified as a function of **L**. Decay rates of the induced EF in Figure 5c,d are represented by the average gradient of profiles in Figure 5a,b.

Figure 5 illustrates the spatial distribution of EF due to various coil combinations. Profiles in Figure 5a depict the decay principles of the EF induced by 4-coil combinations and present a fast-slow variation tendency of EF strength. Among the distances less than 4 cm, the intensities of the EF show an approximately linear decreasing tendency with the increase of **L**. After exceeding the threshold of 4 cm, attenuation rates of the 4-coil combinations gradually slow down and finally, at the depth of 6 cm, reach a stable level of 0.15–0.25. Specifically, different values of CD characterize combinations in the 4-coil group, demonstrating that the coil combination with a high level of CD has a faster attenuation rate compared to the ones with lower CD values. This result can be directly perceived in Figure 5c, which evidently shows the relationship between the value of CD and the field decay rate. When it comes to the 8-coil group, the spatial distribution of EF shows similar behavior to that induced by the 4-coil group. Among the 8-coil combinations, the decay tendency of fast–slow is still visible but less obvious compared to the 4-coil ones. By analyzing Figure 5b,d, we found that coil combination with the highest CD value shows the fastest attenuation rate, whereas the other three configurations illustrate a similar decay property and linearization degree due to comparable CD values. Thus, the general principle that compactly distributed coils can induce an EF with a fast attenuation rate is revealed, and the dispersedly distributed coils show an ability to evoke an EF decaying in a slower way. 4-coil 01 and 8-coil 01 of the smallest CD value in each group have the best performances (maximum depth of the 50% of cortical EF intensity) in penetration depth. At the same time, EF induced by 4-coil 02 and 8-coil 04 generate the lowest depth values at about 3 cm.

It is hard to exactly define the dimension of a square multi-coil array because two important parameters, namely the edge length and the winding size, exist simultaneously and interact with each other. To determine the distribution property of EFs induced by multi-coil arrays of different dimensions, a similar simulation process is applied in coil configurations in Figure 2b–d.

**Figure 6:**
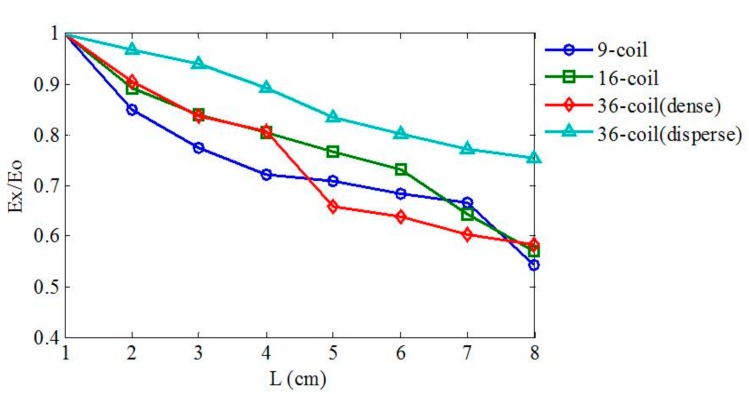
The variation tendency of EF intensity generated by multi-coil arrays of distinct dimensions. The size of an individual coil and an overall spatial distribution of arrays are both influential factors to the variation tendency of the induced EF.

Figure 6 illustrates that different planar arrays of the same topological structure share similar variation properties of induced EF. Profiles in Figure 6 have a variation tendency that decreases relatively faster in a range of **L** and then gradually slows down after a typical value of **L**. With the same spatial overall dimension but a different individual coil size, the 16-coil array, the 36-coil (dense) array, and the 9-coil array have an approximate variation scale ranging from 1 to about 0.55. Furthermore, the EF induced by the 36-coil (disperse) array decays slowly due to the large spatial dimension of stimulating coils. By analyzing profiles in Figure 6, we find that multi-coil arrays of the same overall spatial dimension have a similar penetration ability and verify that the EF attenuation rate depends on the configuration size.

### 3.2. Focality of Coil Combinations

Penetration focality is traditionally regarded as the proportion where the EF intensity exceeds a certain value at a given depth. A high level of EF focality maximizes EF intensity at targeted regions and minimize it at non-interested tissues. To describe and analyze the focality of induced EFs based on different coil combinations, preconditions should be determined first. (1) The stimulating threshold of the EF intensity should be set at a certain value E_T_ (E_T_ = 1 V/cm) [22], and all the EF intensities higher than that should be considered effective values for modulating brain functions. (2) For a certain distance **L**, the energy input should be represented by the corresponding current density that evokes the EF intensity of E_T_ at a specific brain tissue. To estimate the tissue activation ability of induced EF, we quantified the focality by the percentage of cerebral structures exposed to a suprathreshold EF. If the peak intensity of an EF at a target depth exactly reaches the threshold E_T_, then V(θ) represents the proportion between the stimulated volume and the total brain volume [27].

(5) $$V(\theta )=\frac{V_{\Delta }}{V_{M}}$$

where $V_{M}$ is the total volume of the head model and $V_{\Delta }$ represents the overall stimulated volume when the targeted tissue is located at the depth of **L**.

**Figure 7:**
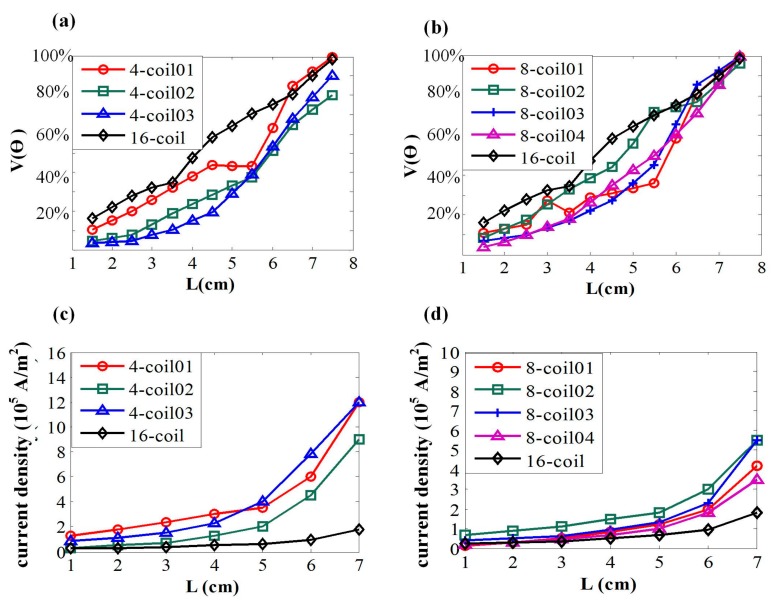
(**a**) Overall activated proportions targeting at different stimulated depths. **L** indicates the length from superficial layer to the interested depth of brain tissue. (**b**) The relationship between targeted depths and activated proportions. (**c**,**d**) Variation tendency of current density with the increase of targeted depth. The input energy is represented by current density of each individual coil.

Figure 7a,b show the stimulated tissue proportions of various targeted depths. For the 4-coil combinations in Figure 7a, all these coil arrays generally follow the rule that values of V(θ) rise with the increase in targeted depths, namely V(θ) has a positive correlation to **L**. Compared to 4-coil 02 and 4-coil 03, coils in 4-coil 01 are arranged dispersedly and a larger value of V(θ) is reached at the same targeted depth, indicating a lower level of coil focality. With a compact arrangement of stimulating coils, 4-coil 03 has the slowest increasing rate of stimulated volume, which can be expected for a better focality in deep regions of the brain. In the range of **L** < 5 cm, 4-coil 03 better stimulates targeted tissues without activating non-interested regions. Based on the simulation results, the correlation between the spatial arrangement of coils and focality is thus shown.

However, when the energy consumption is considered in the selection strategy of coil combinations, more detailed factors need to be included. By analyzing Figure 7a,c comprehensively, 4-coil 02 uses a lower current density to obtain a similar focality of field, which can be considered a more advanced coil selection of the multi-coil array. Similar characteristics of EF are depicted by the profiles in Figure 7b. Based on the fact that more stimulating coils can shape a more complex spatial arrangement, mutual intersects of profiles are generated leading to a relatively less evident distinction among different 8-coil combinations. However, the advantage of a compact arrangement of energized coils can still be shown. 8-coil 04, with the highest level of CD, has the smallest increasing rate of stimulating volume. In the range of **L** > 5.5 cm, 8-coil 04 shows an evident advantage over other combinations among the same group. This result suggests that coil combinations of larger values of CD have a slower increasing rate with the variation of **L**. To better evaluate different 8-coil combinations synthetically, energy consumptions are presented in Figure 7d, showing that compactly distributed coils have superior focality with no sacrifice in energy consumption.

**Figure 8:**
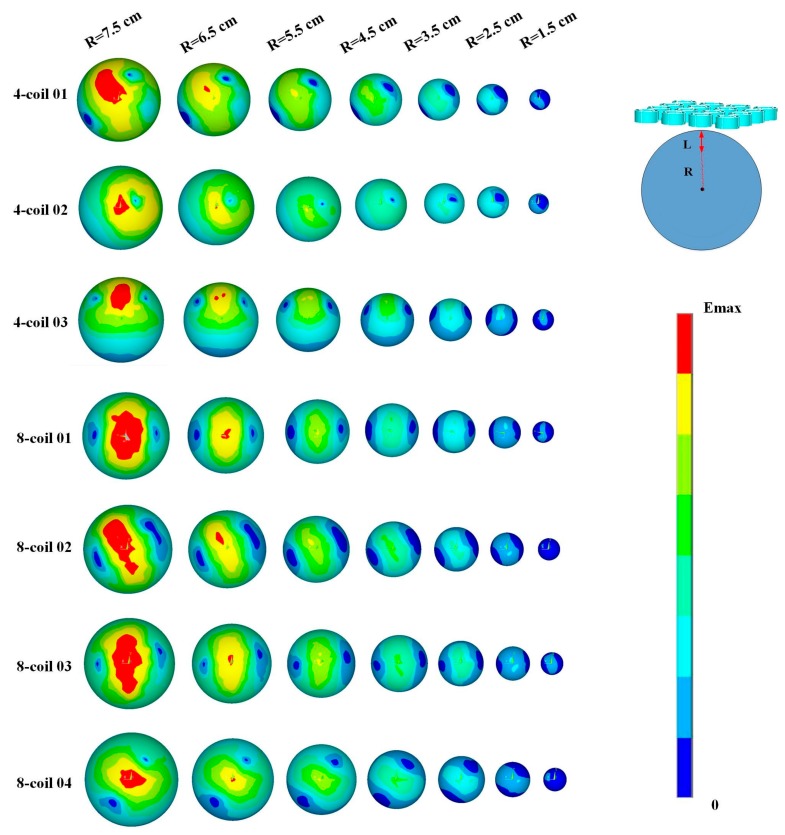
Spatial distribution of the induced EF in different layers of the head model. R meets the relationship of “R = 8.5-**L**”, indicating that R attenuates with the increase of **L**.

The graphs in Figure 8 show the global distribution of induced EFs at different depths of the brain tissue, clearly demonstrating that the geometric form and overall range of an EF greatly depend on the spatial arrangement of energized coils. With higher levels of CD among each groups, 4-coil 02 and 8-coil 04 induce electric fields that have better focality, minimizing side effects on brain tissues. However, with the increase in the targeted depth, the stimulating focality of coil array gradually decays, which generates a large activated region. Moreover, the EF induced by 4-coil 01 distributes in a relatively large area in the center of the head model. The specific region activated by 4-coil 03 is approximately under the energizing coils on account of the asymmetrical structure at the center. 8-coil 01 generates an EF that is parallel to the energizing coils arranged on the edges of the square array. The simulation results in Figure 8 reflect the relationship between the EF distribution and the spatial arrangement of the stimulating coils, providing information for energizing strategies targeted at different brain tissues.

### 3.3. Attenuation-Focality Tradeoff 

**Figure 9:**
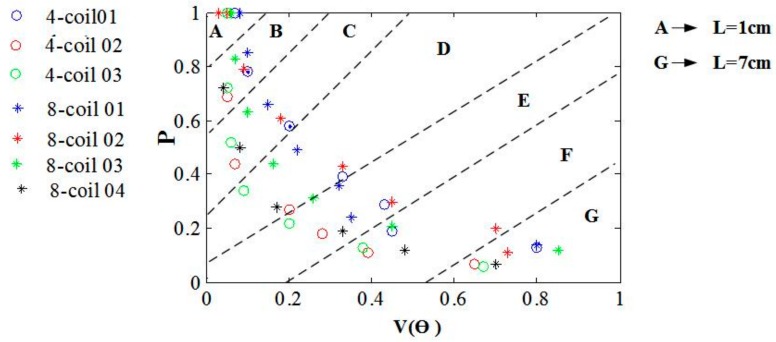
Strengths of the induced EFs at different depth are normalized with respect to the maximum EF amplitude estimated on the cortex. The values of proportion are represented with P. V(θ) shows the activated volume of the head model. The depths of brain tissue are marked A–G, which correspond to **L** from 1 to 7 cm.

By analyzing the decay property of EF intensity and focality of various coil configurations, we gain a result suggesting that there is a tradeoff between these two factors for the aim to stimulate a certain depth of brain tissue. Diverse coil configurations and geometric characteristics induce a significantly different spatial distribution of EF. However, configurations of various stimulating coils are subjected to an attenuation-focality tradeoff that impedes the achievement of a deep and focal stimulation at the same time. In Figure 9, points in the same region marked A–G can stimulate cerebral tissues of a specific depth, depicting the attenuation rate and focality of EFs due to diverse coil combinations. The optimal coil combination is expected to have a minimum V(θ) and a maximum ratio of the EF strength to the intensity of the cortical surface. Peak values of EF intensity at different depths are normalized with respect to the maximum of the EF amplitude estimated on the cortical layer. Points in region A, centered within a small area, represent the EF intensity and stimulating focality of the cortical tissue. The overall characteristics of the points’ distributions show a regular variation tendency with the increase of **L**. From A to G, the points’ projection ranges of V(θ) gradually increase, whereas the projection range of P decreases at the same time. Furthermore, EFs induced by different coil combinations show an obvious diversity of EF intensity but few differences of focality in superficial zones. On the contrary, evident differences in focality and small differences in field intensity are observed in brain tissues of larger depth. In particular, EFs induced by 8-coil combinations have a slower attenuation rate of field intensity, which can be reflected in Regions B–D. However, with the increase in stimulating depth, this advantage of the 8-coil group disappears, and the focality superiority of the 4-coil group gradually becomes apparent. Considering the tradeoff between the attenuation rate and the stimulating focality, it is critical to select a proper coil configuration for different requirements of stimulating depths. In brief, results in Figure 9 can be used as a guideline for the selection of coil configurations that balance the modulation depth and the influence of side effects caused by activation in non-interested tissues.

### 3.4. Target Location by Multi-Coil Arrays

**Figure 10:**
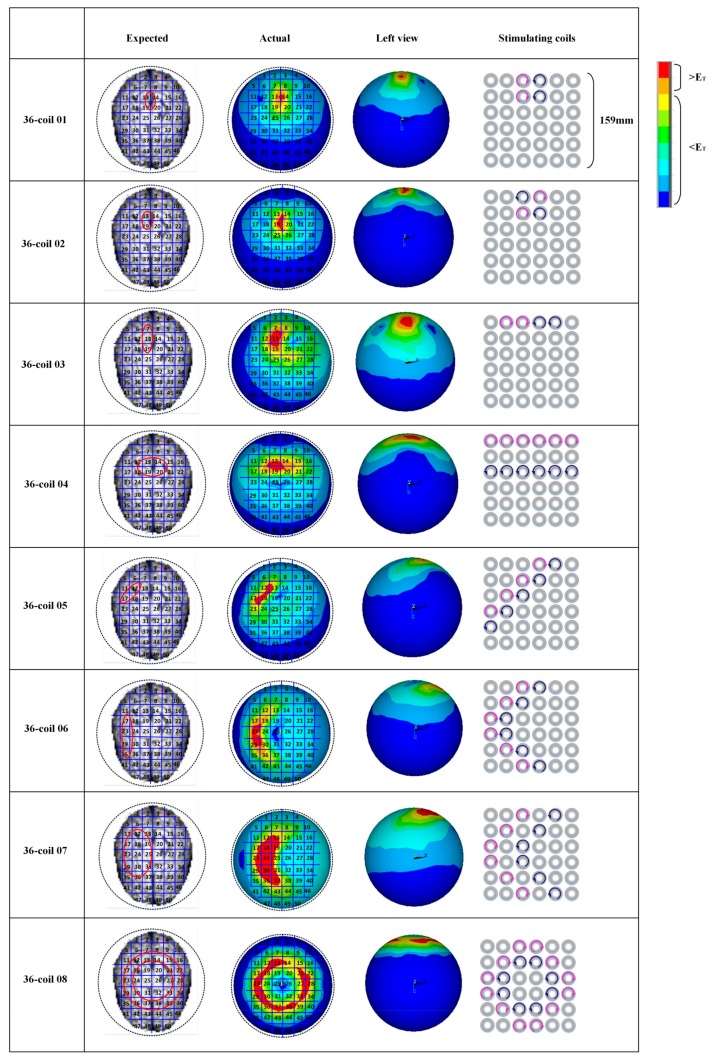
Stimulation results targeted at different cortical tissues. The units in red solid circles are interested regions. The activated parts and energized coils are shown correspondingly. Here, currents in coils are indicated by arrows.

**Figure 11:**
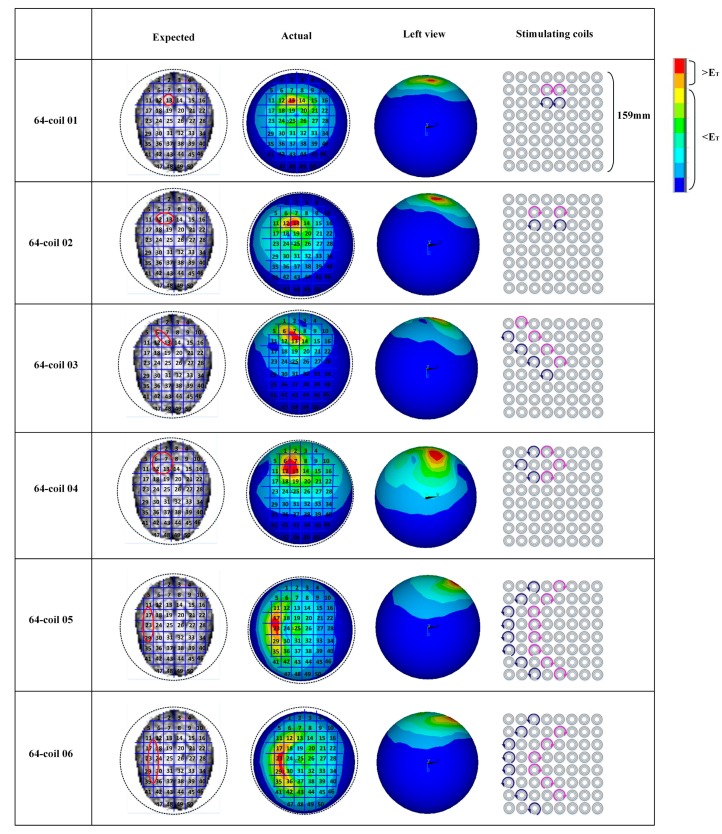
Stimulation results targeted at different cortical tissues. The units in red solid circles are interested regions. The activated parts and energized coils are shown correspondingly. Here, currents in coils are indicated by arrows of different directions.

Figure 10 shows the expected target regions (the second column in Figure 10) and the actually stimulated tissues (the third column in Figure 10) generated by different combinations of energized coils. In each coil array, blue coils are driven by an anticlockwise current and the pink ones are energized by a clockwise current. We here adopt the same threshold value of EF strength (E_T_ = 100 V/m). Any cortical tissue with an EF intensity larger than E_T_ is regarded as the activated region. In order to testify the spatial distribution properties of EF induced by diverse coil combinations and determine feasible control strategies of multi-coil arrays for arbitrary interested tissues, we chose various units in the brain superficial layer as expected target regions and compared these regions with the actually activated parts. Simulation results show that the 36-coil planar array with an edge length of 159 mm and a coil outer radius of 11.9 mm can accurately locate stimulated tissue within an area of 2 units. 36-coil 01 and 36-coil 02 in Figure 10 depict two different interested regions with vertical orientations. We can change the shape of the activated tissue by selecting diverse energizing strategies. The same principle is also described in 36-coil 03 and 36-coil 04. 36-coil 06–08 in Figure 10 show that regions with irregular shapes can also be stimulated by specific coil combinations without activating non-interested tissues. This reduces side effects in TMS treatment. To improve the stimulation accuracy, we designed a 64-coil array with the same edge length of 159 mm and a smaller coil outer radius of 9 mm, as is shown in Figure 11. Results indicate that with a reduced coil size, the 64-coil array can precisely locate the target region within an area of 1 unit, which largely improves the control flexibility and makes it convenient for multi-point stimulation. Comparing 36-coil 06–07 with 64-coil 05–06, we find that the 64-coil array has better stimulating accuracy for interested regions of a similar topological structure, which reduces side effects and improves activating focality.

### 3.5. Comparison of Different Coils

**Figure 12:**
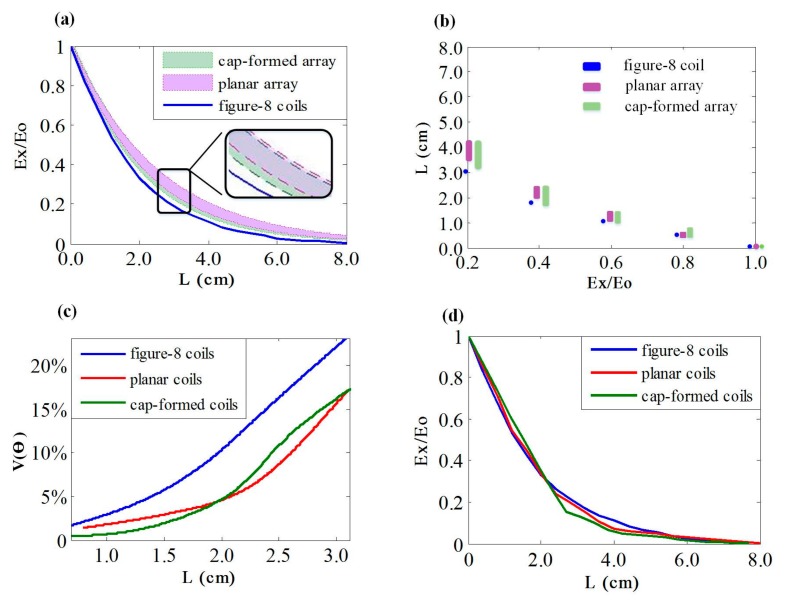
(**a**) Attenuation rate of induced EF in the figure-8 coil, the 36-coil planar array, and the 36-coil cap-formed array. (**b**) Corresponding ranges of activated depth with different coil types. (**c**) The focality of various coils with optimal energizing strategies. (**d**) The attenuation rates of the figure-8 coil, the planar array, and the cap-formed array with optimal energizing strategies.

Figure-8 coils are commonly used in TMS treatment [37]. Attenuation rates and stimulating focality of the figure-8 coil, the 36-coil planar multi-coil array, and the cap-formed array are shown in Figure 12. To describe the attenuation rate of these coils, the peak EF intensity of brain tissues was set to exactly reach the activated threshold. Figure 12a shows that the three types of coils have visibly different attenuation rates of the induced EFs. As the figure-8 coil has a fixed energizing method, the attenuation rate of this coil is depicted by a full line in Figure 12a. Based on diverse energizing strategies, multi-coil arrays show various attenuation rates, which form two specific regions corresponding to the planar array and the cap-formed array, respectively. The EF evoked by the figure-8 coil decays fastest in the range of 0–2 cm and gradually moderates after exceeding the distance of 2 cm. The attenuation property of EF activated by the planar array and the cap-formed array have similar variation tendencies. Figure 12b describes the ranges of activated depth corresponding to diverse attenuation degrees with these three coils. It is shown that the cap-formed array has the largest range of stimulated depth at a certain EF intensity. Both types of the multi-coil array perform better than the figure-8 coil in the attenuation property of induced EFs. Figure 12c depicts the focality of various coils with optimal energizing strategies. As there are diverse energizing methods for multi-coil arrays that can lead to different stimulated volumes, we selected certain methods to activate the smallest brain tissues and compared the results with that activated by the figure-8 coil. It is visibly shown that the figure-8 coil stimulates a larger volume than the planar and the cap-formed arrays at various targeted depths. In the range of 0–2.0 cm, the cap-formed array activates smaller brain tissues and has better focality than the planar form. At the distance of 2.0 cm, V(θ) of both multi-coil arrays reach 5% of the whole head model. With **L** increases, the 36-coil planar array has a better performance of focality until **L** = 3.0 cm. Figure 12d shows the attenuation rates of the figure-8 coil, the planar array, and the cap-formed array with optimal energizing strategies. By analyzing Figure 12c,d, we find that multi-coil arrays can have attenuation properties similar to those of the figure-8 coil and show better focality simultaneously. In general, multi-coil arrays have an attenuation rate of induced EF that is slower than that of figure-8 coils, indicating the improved stimulating depths in TMS. Thus, the multi-coil planar array and the cap-formed array have an advantage over the traditional figure-8 coil in stimulating focality, without sacrificing the attenuation properties of EF intensity.

**Figure 13:**
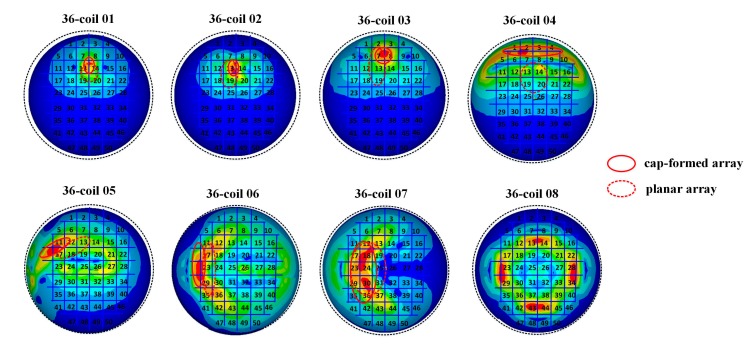
The comparison of the planar array and the cap-formed array. Red full lines indicate cortical regions activated by the selected coils in the cap-formed array. The dashed lines show cortical tissues stimulated by the same corresponding coils in the planar array.

To depict the differences between the planar coil array and the cap-formed array, a further study was made using the same energizing strategies but different coil arrays. All these simulations were completed with the 36-coil cap-formed array. Figure 13 shows the comparison results. The full lines in Figure 13 indicate specific regions activated by the energizing coils in the cap-formed array. The dashed lines depict cortical tissues stimulated by the same corresponding coils in the planar array. Simulation results show that energizing strategies of 36-coil 01, 36-coil 02, and 36-coil 03 can activate smaller regions with the cap-formed array. In addition, the spatial arrangement of coils in a cap-formed array, compared to a planar array, has a more direct impact on the distribution of induced brain tissues Strategies of 36-coil 06, 36-coil 07, and 36-coil 08 demonstrate that regions stimulated by the cap-formed array are farther from the axis perpendicular to the multi-coil array compared to the planar array. For a fixed-shape interested area, cap-formed arrays provide expected stimulus with better controllability.

## 4. Conclusions

In summary, our results suggest that stimulating coils arranged in a disperse form are prone to activating deeper brain regions at the expense of reduced focality. On the contrary, compact distributed coils can evoke an EF with better focality but with fast-decaying intensity. Furthermore, we find that the coil array dimension is significantly related to the attenuation rate of EF intensity, verifying that large multi-coil arrays can activate deeper brain tissues, which satisfies the requirements of TMS. By comprehensively studying the distribution properties of EFs induced by multi-coil arrays, this work provides coil energizing strategies for activating user-defined areas. Results show that a surface area for the cortex of 17 cm^2^ (about 1% of total area) can be accurately stimulated by a 64-coil array with coils 18 mm in diameter, which largely reduces the side effect of TMS. Comparison of the figure-8 coil, the planar array and the cap-formed array demonstrates an improvement in multi-coil configuration in the penetration depth and the focality. This study provides operators with alternative strategies of coil selection and control of the activated regions. Although it is difficult to construct a coil configuration that can achieve a stimulation both deep and focal, the selection and design of proper coil settings can be made on account of a balanced estimation of two factors.

## Figures and Tables

**Table 1:** Coil combinations of 4-coil group and 8-coil group. “a” indicates the distance between adjacent coils in a row, showing values of compact degree (CD) explicitly.

Coil Combinations	Anticlockwise Current	Clockwise Current	CD
4-coil 01	(1) (13)	(4) (16)	$\frac{1}{184.5a^{3}}$
4-coil 02	(6) (10)	(7) (11)	$\frac{1}{6.9a^{3}}$
4-coil 03	(1) (2)	(3) (4)	$\frac{1}{30.0a^{3}}$
8-coil 01	(1) (2) (3) (4)	(13) (14) (15) (16)	$\frac{1}{443.0a^{3}}$
8-coil 02	(1) (2) (9) (13)	(4) (8) (15) (16)	$\frac{1}{454.0a^{3}}$
8-coil 03	(2) (5) (9) (14)	(3) (8) (12) (15)	$\frac{1}{381.0a^{3}}$
8-coil 04	(1) (2) (5) (6)	(11) (12) (15) (16)	$\frac{1}{361.8a^{3}}$
16-coil	(1)–(16)		-

## References

[1] Roth Y., Padberg F., Zangen A. (2007). Transcranial magnetic stimulation of deep brain regions: Principles and methods. Adv. Biol. Psychiatry.

[2] George M.S., Aston-Jones G. (2010). Noninvasive techniques for probing neurocircuitry and treating illness: Vagus nerve stimulation (VNS), transcranial magnetic stimulation (TMS) and transcranial direct current stimulation (tDCS). Neuropsychopharmacology.

[3] Pascual-Leone A., Walsh V., Rothwell J. (2000). Transcranial magnetic stimulation in cognitive neuroscience—Virtual lesion, chronometry, and functional connectivity. Curr. Opin. Neurobiol..

[4] Rastogi P., Lee E.G., Hadimani R.L., Jiles D.C. (2016). Transcranial Magnetic Stimulation-coil design with improved focality. AIP Adv..

[5] Im C.H., Lee C. (2007). Precise Estimation of Brain Electrical Sources Using Anatomically Constrained Area Source (ACAS) Localization. IEEE. Trans. Magn..

[6] Ruohonen J., Ollikainen M., Nikouline V., Virtanen J., Ilmoniemi R.J. (2000). Coil design for real and sham transcranial magnetic stimulation. IEEE Trans. Biomed. Eng..

[7] Starzyn’ski J., Sawicki B., Wincenciak S., Krawczyk A., Zyss T. (2002). Simulation of magnetic stimulation of the brain. IEEE Trans. Magn..

[8] Roth B.J., Cohen L.G., Hallett M.D.M., Friauf M.W., Basser P.J. (1991). Latency of motor evoked potentials to focal transcranial stimulation varies as a function of scalp positions stimulated. Electroencephalogr. Clin. Neurophysiol..

[9] Vahe E.A., Paul J.M., Roger Q.C. (1989). Focal stimulation of human cortex with the magnetic coil: A comparison with electrical stimulation. Exp. Neurol..

[10] Gomez L., Cajko F., Hernandezgarcia L., Grbic A., Michielssen E. (2013). Numerical analysis and design of single-source multicoil TMS for deep and focused brain stimulation. IEEE Trans. Biomed. Eng..

[11] Ueno S., Tashiro T., Harada K. (1988). Localized stimulation of neural tissues in the brain by means of a paired configuration of time-varying magnetic fields. J. Appl. Phys..

[12] Cohen L.G., Roth B.J., Nilsson J., Dang N., Panizza M., Bandinelli S., Friauf W., Hallett M. (1990). Effects of coil design on delivery of focal magnetic stimulation. Tech. Consid. Electroencephalogr. Clin. Neurophysiol..

[13] Nyenhuis J.A., Mouchawar G.A., Bourland J.D., Geddes L.A. (1990). Mouchawar, Influence of coil geometry on localization of the induced electric field in magnetic (eddy-current) stimulation of excitable tissue. IEEE Trans. Magn..

[14] Roth B.J., Saypol J.M., Hallett M., Cohen L.G. (1991). A theoretical calculation of the electric field induced in the cortex during magnetic stimulation. Electroencephalogr. Clin. Neurophysiol..

[15] Rossi S., Hallett M., Rossini P.M., Pascual-Leone A. (2009). Safety, ethical considerations, and application guidelines for the use of transcranial magnetic stimulation in clinical practice and research. Clin. Neurophysiol..

[16] Crowther L.J., Marketos P., Williams P.I., Melikhov Y., Jiles D.C., Starzewski J.H. (2011). Transcranial magnetic stimulation: Improved coil design for deep brain investigation. J. Appl. Phys..

[17] Meng Y., Hadimani R.L., Crowther L.J., Xu Z., Qu J., Jiles D.C. (2015). Deep brain transcranial magnetic stimulation using variable ‘Halo coil’ system. J. Appl. Phys..

[18] Roth Y., Zangen A., Hallett M. (2002). A coil design for transcranial magnetic stimulation of deep brain regions. J. Clin. Neurophysiol..

[19] Deng Z.D., Lisanby S.H., Peterchev A.V. (2013). Electric field depth–focality tradeoff in transcranial magnetic stimulation: Simulation comparison of 50 coil designs. Brain Stimul..

[20] Deng Z.D., Lisanby S.H., Peterchev A.V. (2014). Coil design considerations for deep transcranial magnetic stimulation. Clin. Neurophysiol..

[21] Ruohonen J., Ravazzani P., Grandori F., Ilmoniemi R.J. (1999). Theory of multichannel magnetic stimulation: Toward functional neuromuscular rehabilitation. IEEE Trans. Biomed. Eng..

[22] Im C.H., Lee C. (2006). Computer-Aided Performance evaluation of a multichannel transcranial magnetic stimulation system. IEEE Trans. Magn..

[23] Cline C.C., Johnson N.N., He B. Subject-specific optimization of channel currents for multichannel transcranial magnetic stimulation. Proceedings of the 2015 37th Annual International Conference of the IEEE Engineering in Medicine and Biology Society (EMBC).

[24] Laakso I., Hirata A., Ugawa Y. (2014). Effects of coil orientation on the electric field induced by TMS over the hand motor area. Phys. Med. Biol..

[25] Thielscher A., Kammer T. (2004). Electric field properties of two commercial figure-8 coils in TMS: Calculation of focality and efficiency. Clin. Neurophysiol..

[26] Janssen M., Oostendorp T.F., Stegeman D.F. (2015). The coil orientation dependency of the electric field induced by TMS for M1 and other brain areas. J. Neuroeng. Rehabil..

[27] Deng Z.D. (2013). Electromagnetic Field Modeling of Transcranial Electric and Magnetic Stimulation: Targeting, Individualization, and Safety of Convulsive and Subconvulsive Applications.

[28] Bersani F.S., Minichino A., Enticott P.G., Mazzarini L., Khan N., Antonacci G. (2013). Deep transcranial magnetic stimulation as a treatment for psychiatric disorders: A comprehensive review. Eur. Psychiatry.

[29] Lee W.H., Lisanby S.H., Laine A.F., Peterchev A.V. (2016). Comparison of electric field strength and spatial distribution of electroconvulsive therapy and magnetic seizure therapy in a realistic human head model. Eur. Psychiatry.

[30] Rochelabarbe N., Aarabi A., Kongolo G., Gondryjouet C., Dümpelmann M., Grebe R., Wallois F. (2008). High-resolution electroencephalography and source localization in neonates. Hum. Brain Mapp..

[31] Roth Y.G., Pell S., Zangen A. (2014). Realistic shape head model and spherical model as methods for TMS coil characterization. Clin. Neurophysiol..

[32] Guadagnin V., Parazzini M., Fiocchi S. (2015). Deep transcranial magnetic stimulation: modeling of different coil configurations. IEEE Trans. Biomed. Eng..

[33] Gao F., Zhang F.S., Wakatsuchi H., Sievenpiper D.F. (2015). Synthesis and design of programmable subwavelength coil array for near-field manipulation. IEEE Trans. Microw. Theory Tech..

[34] Windhoff M., Opitz A., Thielscher A. (2011). Electric field calculations in brain stimulation based on finite elements: An optimized processing pipeline for the generation and usage of accurate individual head models. Hum. Brain Mapp..

[35] Mcgirr A., Eynde F.V.D., Chachamovich E., Fleck M.P.A., Berlim M.T. (2014). Personality dimensions and deep repetitive transcranial magnetic stimulation (dtms) for treatment-resistant depression: A pilot trial on five-factor prediction of antidepressant response. Neurosci. Lett..

[36] Lin V.W., Hsiao I.N., Dhaka V. (2000). Magnetic coil design considerations for functional magnetic stimulation. IEEE Trans. Biomed. Eng..

[37] Iwahashi M., Gomeztames J., Laakso I., Hirata A. (2017). Evaluation method for in situ electric field in standardized human brain for different transcranial magnetic stimulation coils. Phys. Med. Biol..

